# Efficacy and safety of hepatic artery infusion chemotherapy combined with tyrosine kinase inhibitors plus programmed death-1 inhibitors for hepatocellular carcinoma refractory to transarterial chemoembolization

**DOI:** 10.3389/fonc.2023.1178428

**Published:** 2023-05-03

**Authors:** Long-Wang Lin, Kun Ke, Le-Ye Yan, Rong Chen, Jing-Yao Huang

**Affiliations:** Department of Interventional Radiology, Fujian Medical University Union Hospital, Fuzhou, China

**Keywords:** lenvatinib, refractory to TACE, programmed death-1 inhibitor, hepatic artery infusion chemotherapy, hepatocellular carcinoma

## Abstract

**Background:**

The subsequent therapy for hepatocellular carcinoma (HCC) patients with refractory to transarterial chemoembolization (TACE) is still controversial. This study was performed to evaluate the efficacy and safety of combination therapy comprising hepatic artery infusion chemotherapy (HAIC), lenvatinib, and programmed death-1 inhibitors relative to HAIC combined with lenvatinib.

**Methods:**

In this single-center retrospective study, we analyzed data from HCC patients with refractory to TACE from June 2017 to July 2022. Primary study outcomes were overall survival (OS) and progression-free survival (PFS), while the secondary outcomes were the objective response rate (ORR), disease control rate (DCR), and treatment-related adverse events.

**Results:**

We enrolled 149 patients finally, including 75 patients who received HAIC combined with lenvatinib plus PD-1 inhibitors therapy (HAIC+L+P group) and 74 patients who received HAIC combined with lenvatinib therapy (HAIC+L group). The median OS in the HAIC+L+P group (16.0; 95% CI: 13.6~18.3 months) was significantly higher compared to the HAIC+L group (9.0; 95% CI: 6.5~11.4 months) (*p* = 0.002), while the median PFS in the HAIC+L+P group (11.0; 95% CI: 8.6~13.3 months) was significantly higher compared to the HAIC+L group (6.0; 95% CI: 5.0~6.9 months) (*p* < 0.001). Significant between-group differences in DCR (*p* = 0.027) were found. Additionally, 48 pairs of patients were matched after propensity matching analysis. The survival prognosis between two groups before propensity matching is similar to that after propensity matching. Moreover, the percentage of patients with hypertension in the HAIC+L+P group was significantly higher compared to the HAIC+L group (28.00% vs. 13.51%; *p* = 0.029).

**Conclusions:**

A combination therapy of HAIC, lenvatinib, and programmed death-1 inhibitors significantly improved oncologic response and prolonged survival duration, showing a better survival prognosis for HCC patients with refractory toTACE.

## Introduction

Hepatocellular carcinoma (HCC) has the third highest mortality rate and is the sixth most common malignancy worldwide (1). Approximately 70% of new cases have the disease diagnosed at an advanced stage, which makes them ineligible for surgical resection (2). According to the Barcelona Clinic Liver Cancer (BCLC) clinical staging system, transarterial chemoembolization (TACE) is recommended as first-line therapy for HCC patients in BCLC stage B (3). Compared with supportive therapy, TACE significantly improved clinical outcomes and provided a survival benefit (4). However, TACE frequently results in incomplete tumor necrosis, eventually becoming less effective (5). Furthermore, as the number of TACE increases, the efficacy of repeated TACE is significantly reduced (6). The Liver Society of Japan (JSH) and the Liver Cancer Study Group of Japan (LCSGJ) define this phenomenon as “TACE refractoriness.” TACE refractoriness is associated with poor prognosis (7).

Although there are no widely accepted treatment guidelines for TACE failure or refractory treatment (8), therapies such as hepatic artery infusion chemotherapy (HAIC), tyrosine kinase inhibitors (TKI), and programmed death-1 (PD-1) inhibitors are all potentially available for TACE failure or refractory treatment (9–11).

HAIC or TKI such as sorafenib and lenvatinib are widely used as alternative therapies for TACE failure or refractory treatment (12, 13). Previously published clinical studies have shown that HAIC may be effective, as evidenced by improving the long-term survival prognosis for HCC patients with TACE refractory (9). In addition, switching to a tyrosine kinase inhibitor significantly improved treatment response rates and overall survival in TACE-refractory patients compared with continuing TACE (14). TKI has been recommended as standard treatment for TACE resistance (15). A recent study showed that overall survival (OS) and progression-free survival (PFS) in TACE-refractory patients were prolonged by switching the treatment to sorafenib and lenvatinib (15, 16). However, the efficacy of HAIC or TKI monotherapy remains unsatisfactory in patients with TACE failure or refractory (17). Combination therapy has been considered (18).

As another possible subsequent choice of therapy for TACE resistance, immunotherapy with PD-1 inhibitors has been shown to be associated with survival benefits (19). Specifically, for these HCC patients, data from a clinical trial showed stereotactic body radiation therapy combined with a PD-1 inhibitor improves PFS and OS compared to continuing TACE in TACE-refractory patients (20). Another study also showed that the combination of HAIC plus PD-1 inhibitors prolonged PFS and OS in advanced HCC (21). Moreover, TACE therapy combined with TKI plus PD-1 inhibitors showed great promise in improving clinical prognosis for HCC patients with refractory to TACE (19).

Although there are no additional effective treatment choices for HCC patients with refractory to TACE thus far, many researchers are making great efforts to confirm the most effective combination therapy (22). Up to now, there have been little data on HAIC combined with TKI plus PD-1 inhibitors for HCC refractory to TACE. We hypothesize that combining HAIC with lenvatinib and PD-1 inhibitors may bring new ideas for subsequent treatment in HCC patients who are refractory to TACE. This combination therapy may be synergistic in its antitumor effects and help improve survival outcomes of patients with HCC refractory to TACE. Therefore, this study aimed to determine the effectiveness and safety of this combination therapy in TACE-refractory HCC patients by comparing it with treatment with HAIC plus lenvatinib.

## Materials and methods

### Patients

**Figure 1:**
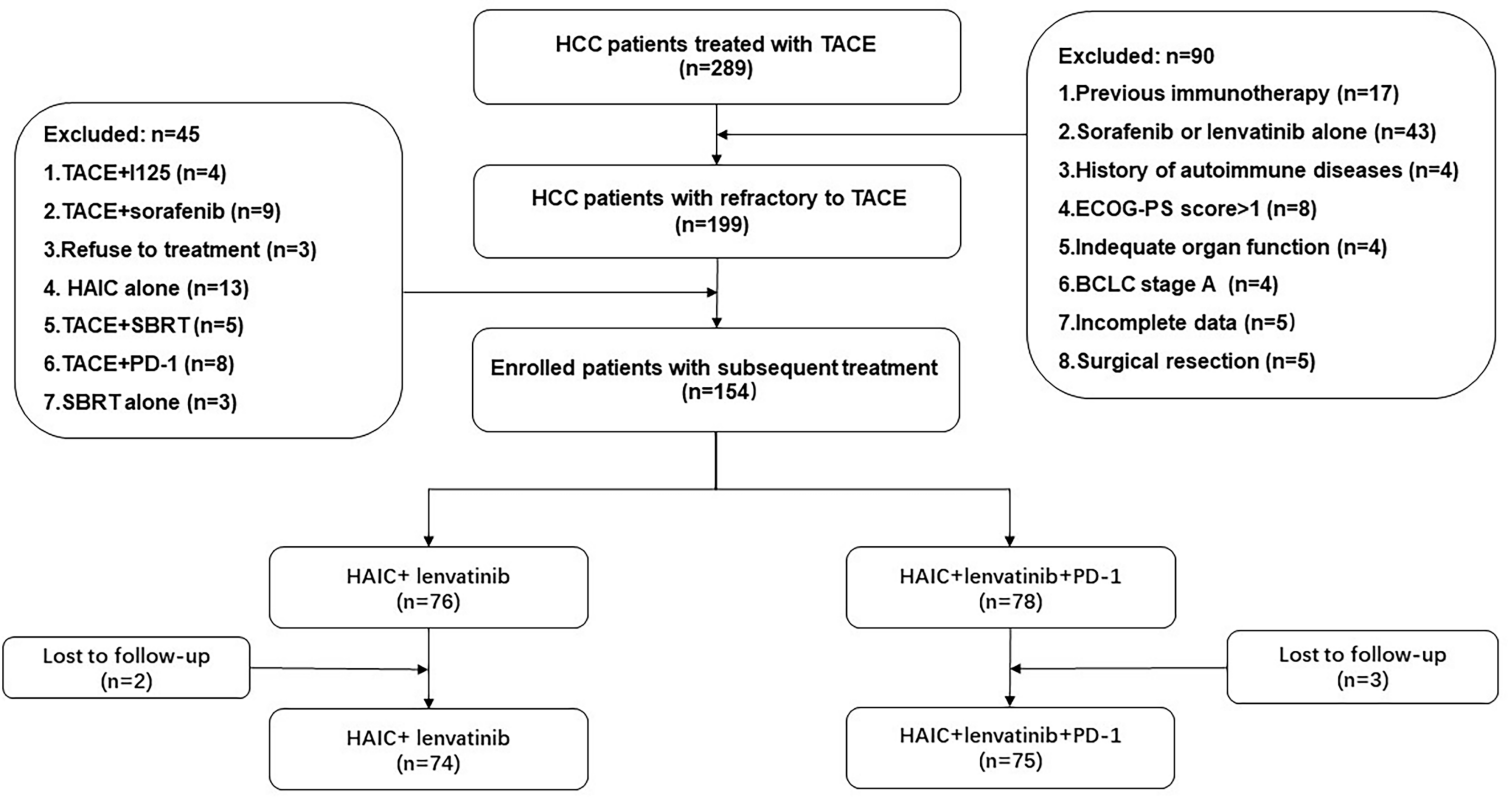
Flowchart of the patient selection process. HCC, hepatocellular carcinoma; ECOG-PS, Eastern Cooperative Oncology Group Performance Status; BCLC, Barcelona Clinic Liver Cancer; TACE, transarterial chemoembolization; HAIC, hepatic artery infusion chemotherapy; PD-1, programmed cell death-1.

The study initially evaluated 289 HCC patients from June 2017 to July 2022. The screening process is shown in **Figure 1**. A total of 149 HCC patients with HCC refractory to TACE were finally included in this study, stratified as 75 patients treated with the combination treatment of HAIC, lenvatinib, and PD-1 inhibitors (HAIC+L+P group) and 74 patients treated with the treatment of HAIC combined with lenvatinib (HAIC+L group).

The inclusion criteria were as follows: (1) unresectable HCC; (2) receipt of therapy (HAIC combined with lenvatinib therapy or the combination treatment of HAIC, lenvatinib, and PD-1 inhibitors); (3) BCLC stage B/C; (4) Eastern Cooperative Oncology Group Performance Status (ECOG-PS) score ≤ 1; (5) Child–Pugh class A or B; and (6) TACE failure or refractory.

The exclusion criteria were as follows: (1) patients with severe autoimmune diseases; (2) patients receiving previous systemic or immunotherapy; (3) patients with incomplete data; (4) patients receiving other tyrosine kinase inhibitor treatment; (5) patients with ECOG-PS score > 1; (6) patients refusing treatment; (7) patients with other subsequent treatments; (8) contraindications for HAIC, lenvatinib, PD-1 inhibitors; and (9) patients lost to follow-up.

The data at baseline were recorded, including age, sex, etiology of cirrhosis, Child–Pugh score, cirrhosis, BLCL stage, Cheng’s portal vein tumor thrombosis (PVTT) classification, tumor numbers, largest tumor size in diameter, extrahepatic metastasis, alpha-fetoprotein (AFP), carbohydrate antigen 199 (CA199), arterioportal fistulas (APFs), prothrombin time (PT), albumin (ALB), total bilirubin (TBIL), aspartate aminotransferase (AST), alanine aminotransferase (ALT), alkaline phosphatase (ALP), and γ-glutamyl transferase (GGT). Cheng’s PVTT classification was used as previously described (23).

### Treatment option

The therapy options were stratified as HAIC combined with lenvatinib and combination treatment of HAIC, lenvatinib, and PD-1 inhibitors. The patient’s wishes and the clinician’s decisions were considered when determining the treatment plan.

### Definition of TACE failure/refractoriness

The 2014 JSH–LCSGJ criteria were used to define TACE failure/refractoriness (7, 24): (1) consecutive progression in the liver after adequate implementation of selective TACE, even after replacement of chemotherapeutic agents and reanalysis of feeding arteries seen on response evaluation CT/MRI at 1–3 months (compared to a previous number of tumors before the TACE procedure); (2) two or more consecutive inadequate responses (surviving lesions >50%) in treated tumors after adequate implementation of selective TACE, even after replacement of chemotherapeutic agents and reanalysis of feeding arteries seen on response evaluation CT/MRI at 1–3 months; (3) persistent elevation of tumor contrast immediately after TACE, even when a slight temporary decrease is observed; (4) presence of vascular invasion and (5) presence of extrahepatic spread.

### HAIC

HAIC was performed by experienced physicians. HAIC was conducted on 1~2 days as previous data reported. The catheter is inserted into the femoral trunk or superior mesenteric artery for arteriography to observe the blood supply to the tumor. The microcatheter was then fixed in the main tumor supply artery after being super-selectively inserted and positioned to return to the ward for perfusion chemotherapy. FOLFOX-HAIC was performed for a 3-week cycle regimen. Using the microcatheter, chemotherapy agent was infused as follows: oxaliplatin is dosed at 85 mg/m^2^ between hours 0 and 2; leucovorin is dosed at 400 mg/m^2^ between hours 2 and 3; and 5-fluorouracil is dosed at 2,400 mg/m^2^ over 23 or 46 h.

### Lenvatinib and PD-1 inhibitors

On the fourth to sixth days after HAIC, lenvatinib (4 mg/pill) was administered orally at a dosage of 12 mg (when the body weight is over 60 kg) or 8 mg (when the body weight is below 60 kg) daily. In the event of grade 3 or 4 treatment-related adverse events (TRAEs), the lenvatinib dose was reduced to a dosage of 8 mg (when the body weight is over 60 kg) or 4 mg (when the body weight is below 60 kg) daily.

On the fourth to sixth days after HAIC, a PD-1 inhibitor was administered simultaneously. PD-1 inhibitors (camrelizumab (200 mg/bottle) or sintilimab (100 mg/bottle)) were administered intravenously at a dosage of 200 mg for 3 weeks and then stopped for 1 week. Every 4 weeks comprises a treatment cycle. Corticosteroids were used when severe immune-related TRAEs occurred.

After adjustment, as we described above, lenvatinib and PD-1 inhibitors were discontinued when grade 3 or 4 TRAEs continued. The dosage was recovered when the toxicity was diminished or the patient could tolerate the treatment (according to the discretion of the investigator).

### Treatment evaluation and follow-up

The primary outcomes were OS and PFS. OS was defined as the time interval from the initiation of the HAIC treatment to the patient’s death, while PFS was defined as the time interval from the initiation of the HAIC treatment to the first documentation of disease progression or the patient’s death. Additionally, the Modified Response Evaluation Criteria in Solid Tumors (mRECIST) was used for treatment response (25), which included complete response (CR), partial response (PR), stable disease (SD), and progression disease (PD). The DCR was defined as the sum of CR, PR, and SD. The ORR was defined as the sum of CR and PR. Patients were followed up every 5 to 7 weeks to monitor disease status with imaging examination (computed tomography or magnetic resonance imaging). TRAEs were assessed using the Common Terminology Criteria for Adverse Events version 5.0.

### Propensity score matching analysis

To decrease bias in the selection of patients, the propensity score matching (PSM) analysis was carried out between the HAIC+T+P and the HAIC+T groups. In our model, we matched variables showing significant differences or associations with patient selection, such as age, sex, etiology of cirrhosis, Child–Pugh score, cirrhosis, BLCL stage, Cheng’s PVTT classification, tumor numbers, largest tumor size in diameter, and extrahepatic metastasis. The value of the caliper was 0.03 when one-to-one matching was applied without replacement.

### Statistical analysis

All statistical analyses were conducted using SPSS software version 25.0 (IBM, Chicago, IL, USA) and Prism 8 (GraphPad Software, San Diego, CA, USA). Categorical variables were expressed using numbers and percentages (*n* (%)), and continuous variables were displayed utilizing the mean with standard deviation (mean ± standard deviation) or median (interquartile range) based on the normality of data. Categorical variables were compared using the Chi-square test, and continuous variables were compared utilizing an independent Mann–Whitney *U* test or sample *t*-test based on the normality of data. The survival curve analysis was evaluated by the Kaplan-Meier method, and differences were performed utilizing the log-rank test. The Cox proportional hazards model was applied to univariate and multivariate analyses for OS and PFS. The univariable Cox proportional hazards model was included in each variable; then, the variables with a two-sided *p*-value of < 0.05 were fitted in the multivariable analysis, with a stepwise Cox hazard regression model used to identify their value as independent predictors of OS and PFS. *p* < 0.05 was considered statistically significant.

## Results

### Baseline characteristics

The characteristics of the enrolled 149 patients are shown. No statistically significant difference between the two groups was found with regard to age (*p* = 0.692), sex (*p* = 0.242), etiology (*p* = 0.106), Child–Pugh score (*p* = 0.678), BLCL stage (*p* = 0.688), cirrhosis (*p* = 0.444), Cheng’s PVTT classification (*p* = 0.816), largest tumor size (*p* = 0.230), tumor numbers (*p* = 0.982), AFP level (*p* = 0.562), CA199 (*p* = 0.532), extrahepatic metastases (*p* = 0.178), APFs (*p* = 0.463), ascites (*p* = 0.901), TBIL (*p* = 0.903), ALB (*p* = 0.338), ALT (*p* = 0.307), AST (*p* = 0.093), GGT (0.140), and ALP (*p* = 0.239). The median number of HAIC courses per patient was 3.91 ± 1.47 in the HAIC+L+P group compared with 3.69 ± 1.18 in the HAIC+L group (*p* = 0.586). The baseline characteristics of patients between the two groups before propensity matching are similar to that after propensity matching (**Table 1**).

**Table 1:** Baseline characteristics of patients.

Characteristics	Before PSM HAIC+L+P (*n* = 75)	HAIC+L (*n* = 74)	*p*-value	After PSM HAIC+L+P (*n* = 48)	HAIC+L (*n* = 48)	*p*-value
Age (years)	55.3 ± 9.5	56.0 ± 10.5	0.692	55.1 ± 10.0	54.5 ± 10.4	0.780
Sex (*n* (%))			0.242			0.765
Male	66 (88.00%)	60 (81.10%)		41 (85.42%)	42 (87.50%)	
Female	9 (12.00%)	14 (18.90%)		7 (14.58%)	6 (12.50%)	
Etiology (*n* (%))			0.106			0.435
Hepatitis B	70 (93.33%)	63 (85.13%)		43 (89.59%)	46 (95.84%)	
Hepatitis C	0 (0.00%)	0 (0.00%)		0 (0.00%)	0 (0.00%)	
Nonhepatitis B and C	5 (6.670%)	11 (14.87%)		5 (10.41%)	2 (4.16%)	
Child–Pugh score (*n* (%))			0.678			1.000
5–6	55 (73.33%)	52 (70.27%)		36 (75.00%)	36 (75.00%)	
7–9	20 (26.67%)	22 (29.73%)		12 (25.00%)	12 (25.00%)	
BLCL stage			0.688			0.679
B	44 (58.67%)	41 (55.41%)		27 (56.25%)	29 (60.42%)	
C	31 (41.33%)	33 (44.59%)		21 (43.75%)	19 (39.58%)	
Cirrhosis (*n* (%))			0.444			0.232
Present	51 (68.00%)	53 (71.62%)		34 (70.84%)	39 (81.25%)	
Absent	24 (32.00%)	21 (28.38%)		14 (29.16%)	9 (18.75%)	
Cheng’s PVTT classification			0.816			0.679
Present	28 (37.33%)	29 (39.18%)		27 (56.25%)	29 (60.42%)	
Absent	47 (62.67%)	45 (60.82%)		21 (43.75%)	19 (39.58%)	
Largest tumor size (cm, in diameter)	8.3 ± 3.8	7.5 ± 3.8	0.230	8.0 ± 3.6	7.9 ± 3.8	0.890
Tumor numbers (*n* (%))			0.982			1.000
>3	70 (93.33%)	69 (93.24%)		44 (91.67%)	45 (93.75%)	
≤3	5 (6.67%)	5 (6.76%)		4 (8.33%)	3 (6.25%)	
AFP (ng/ml)			0.562			0.682
>400	37 (49.33%)	33 (44.54%)		21 (43.75%)	23 (47.91%)	
≤400	38 (50.67%)	41 (55.46%)		27 (56.25%)	25 (52.08%)	
CA199 (U/ml)	25.3 (14.9~52.3)	28.5 (15.1~55.0)	0.532	23.1 (19.1~35.5)	27.3 (12.8~48.1)	0.424
Extrahepatic metastases (*n* (%))			0.178			0.386
Present	28 (37.34%)	20 (27.03%)		18 (37.50%)	14 (29.16%)	
Absent	47 (62.66%)	54 (72.97%)		30 (62.50%)	34 (70.84%)	
APFs (*n* (%))			0.463			0.411
Present	30 (40.00%)	34 (45.94%)		19 (39.58%)	23 (47.91%)	
Absent	45 (60.00%)	40 (54.06%)		29 (60.42%)	25 (52.09%)	
Ascites			0.901			0.824
Present	23 (30.66%)	22 (29.72%)		14 (29.16%)	15 (31.25%)	
Absent	52 (69.34%)	52 (70.28%)		34 (60.84%)	33 (68.75%)	
TBIL (µmol/L)	18.6 (12.6~29.3)	18.3 (11.9~29.2)	0.903	19.1 (12.6~27.6)	16.8 (11.8~26.3)	0.603
ALB (g/L)	35.9 ± 6.3	34.8 ± 5.7	0.338	36.3 ± 4.6	37.0 ± 5.8	0.517
ALT (IU/L)	45.2 (27.0~58.6)	37.5 (27.0~52.2)	0.307	45.0 (29.0~59.0)	36.0 (28.5~48.5)	0.103
AST (IU/L)	71.0 (42.4~110.1)	52.5 (36.2~87.3)	0.093	68.5 (42.5~100.0)	51.0 (35.0~82.5)	0.476
GGT (IU/L)	198.2 (116.2~338.1)	159.4 (176.8~336.4)	0.140	203.1 (10.2~305.1)	152.0 (74.2~331.7)	0.216
ALP (IU/L)	182.2 (125.6~267.2)	147.4 (111.5~242.8)	0.239	180.2 (125.6~253.2)	140.2 (103.2~216.6)	0.086
HAIC courses	3.91 ± 1.47	3.69 ± 1.18	0.586	4.08 ± 1.58	3.81 ± 1.25	0.562
2	7 (9.33%)	7 (9.45%)		3 (6.25%)	3 (6.25%)	
3	34 (45.33%)	35 (47.29%)		21 (43.75%)	24 (50.00%)	
4	11 (14.66%)	14 (18.91%)		9 (18.75%)	7 (14.51%)	
5	13 (17.33%)	12 (16.21%)		7 (14.51%)	9 (18.75%)	
6	5 (6.66%)	4 (5.40%)		3 (6.25%)	3 (6.25%)	
7	2 (2.66%)	2 (2.70%)		2 (4.16%)	2 (4.16%)	
8	3 (4.00%)	0 (0.00%)		3 (6.25%)	0 (0.00%)	

Data are presented as n (%), mean ± standard deviation, and median (interquartile range). HAIC, hepatic artery infusion chemotherapy; HBV, hepatitis B virus; BCLC, Barcelona Clinic Liver Cancer. PVTT, portal vein tumor thrombosis; AFP, alpha-fetoprotein; CA199, carbohydrate antigen 199; APFs, arterioportal fistulas; PT, prothrombin time; ALB, albumin; TBIL, total bilirubin; AST, aspartate aminotransferase; ALT, alanine aminotransferase; GGT, γ-glutamyl transferase; ALP, alkaline phosphatase.

### OS and PFS

**Figure 2:**
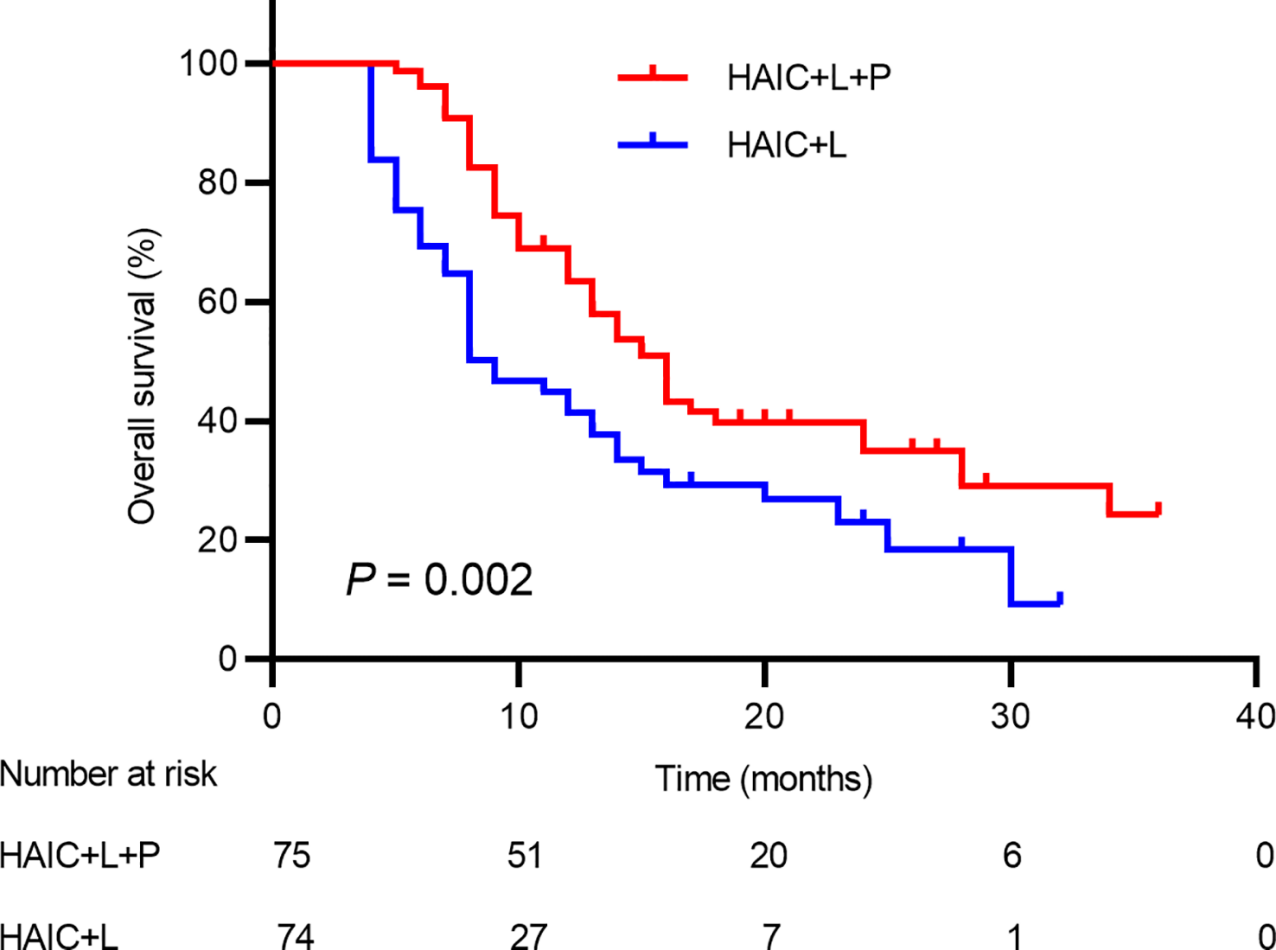
Kaplan–Meier analysis of overall survival in patients receiving the combination therapy of HAIC, lenvatinib plus PD-1 inhibitor, and HAIC plus lenvatinib therapy. HAIC, hepatic artery infusion chemotherapy; PD-1, programmed cell death-1.

There were 97 deaths during the follow-up period, including 48 deaths (64.00%) in the HAIC+T+P group and 49 deaths (66.21%) in the HAIC+T group. The median OS in the HAIC+T+P group (16.0; 95% CI: 13.6~18.3 months) was significantly higher compared to the HAIC+T group (9.0; 95% CI: 6.5~11.4 months) (*p* = 0.002) (**Figure 2**). Additionally, 48 pairs of patients were matched after propensity matching analysis. The median OS between the two groups before propensity matching is similar to that after propensity matching (**Supplementary Figure S1**).

**Figure 3:**
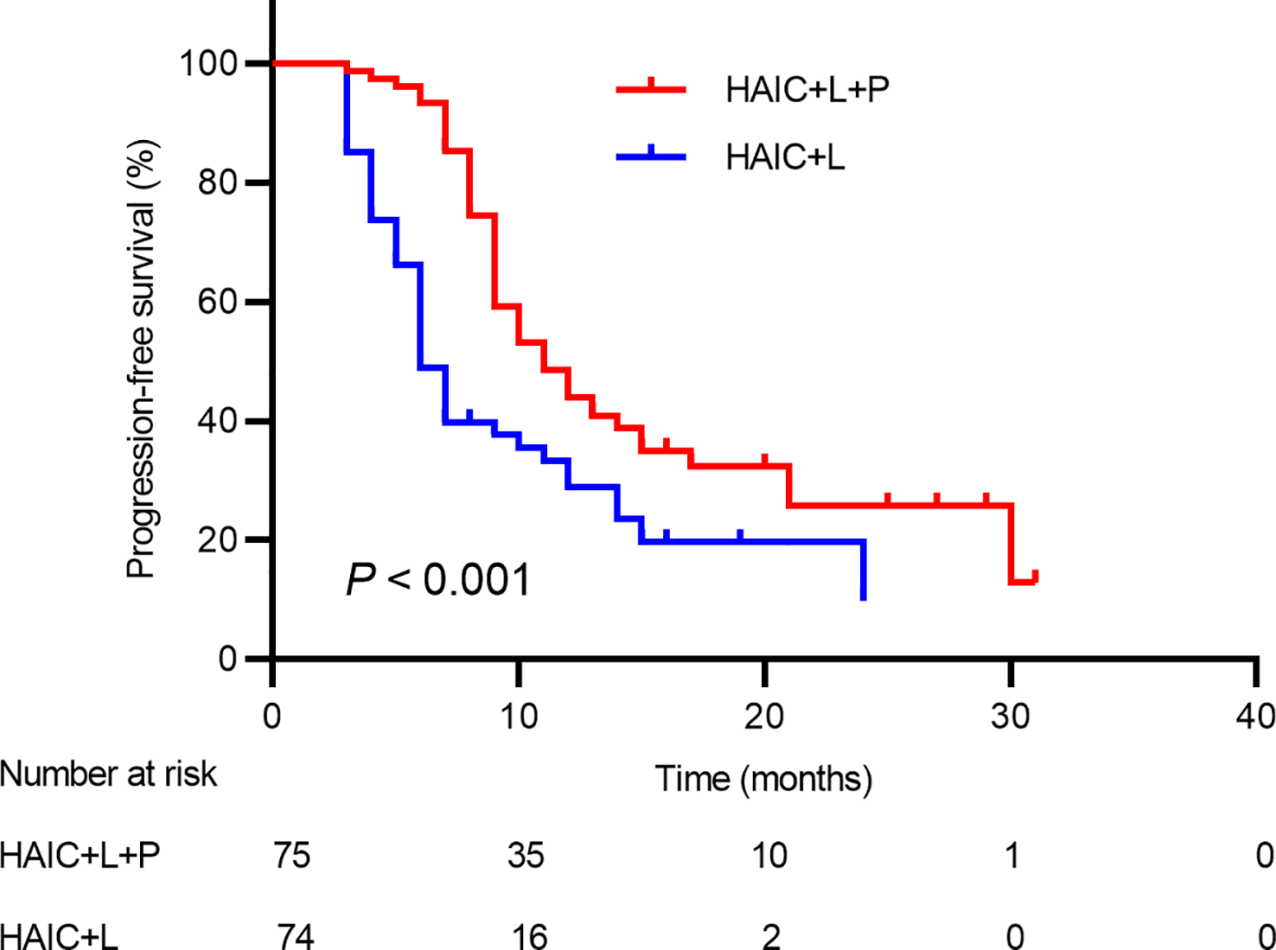
Kaplan-Meier analysis of progression-free survival in patients receiving the combination therapy of HAIC, lenvatinib plus PD-1 inhibitor, and HAIC plus lenvatinib therapy. HAIC, hepatic artery infusion chemotherapy; PD-1, programmed cell death-1.

Tumor progression was observed in 44 patients in total, including 16 patients (21.33%) in the HAIC+T+P group and 28 patients (37.83%) in the HAIC+T group. The median PFS in the HAIC+T+P group (11.0; 95% CI: 8.6~13.3 months) was significantly higher compared to the HAIC+T group (6.0; 95% CI: 5.0~6.9 months) (*p* < 0.001) (**Figure 3**). Additionally, 48 pairs of patients were matched after propensity matching analysis. The median PFS between the two groups before propensity matching is similar to that after propensity matching (**Supplementary Figure S2**).

The duration of treatment can be reflected by the HAIC course. Although there was a progressive tendency for patients to have longer OS with more sessions of HAIC treatment, there was no significant difference (*p* = 0.433) (**Supplementary Figure S3**). Furthermore, although there was a progressive tendency for patients to have longer PFS with more sessions of HAIC treatment, there was no significant difference (*p* = 0.483) (**Supplementary Figure S4**).

### Treatment response

According to the mRECIST criteria, one patient (1.33%) in the HAIC+T+P group and one patient (1.35%) in the HAIC+T group achieved CR (**Supplementary Figures S5, 6**); 33 patients (44.00%) in the HAIC+T+P group and 24 patients (32.43%) in the HAIC+T group achieved PR; 25 patients (33.33%) in the HAIC+T+P group and 21 patients (28.37%) in the HAIC+T group achieved SD; 16 patients (21.33%) in the HAIC+T+P group and 28 patients (37.83%) in the HAIC+T group had PD; 34 patients (45.33%) in the HAIC+T+P group and 25 patients (33.78%) in the HAIC+T group achieved an objective response; and 59 patients (78.66%) in the HAIC+T+P group and 46 patients (62.16%) in the HAIC+T group achieved disease control. Significant differences between the two groups were revealed in DCR (*p* = 0.027); however, no significant differences between the two groups were revealed in ORR (*p* = 0.146) (**Table 2**). Additionally, 48 pairs of patients were matched after propensity matching analysis. Treatment response between two groups before propensity matching is similar to that after propensity matching (**Supplementary Table S1**).

**Table 2:** Treatment response as assessed by imaging features according to the mRECIST criteria in two groups.

Curative effect	HAIC+L+P	HAIC+L	*p*-value
Complete response (CR)	1 (1.33%)	1 (1.35%)	0.992
Partial response (PR)	33 (44.00%)	24 (32.43%)	0.114
Stable disease (SD)	25 (33.33%)	21 (28.37%)	0.445
Progressive disease (PD)	16 (21.33%)	28 (37.83%)	0.027
Overall response rate (ORR)	34 (45.33%)	25 (33.78%)	0.146
Disease control rate (DCR)	59 (78.66%)	46 (62.16%)	0.027

mRECIST, Modified Response Evaluation Criteria in Solid Tumors; HAIC, hepatic artery infusion chemotherapy.

### Factors associated with OS and PFS

**Figure 4:**
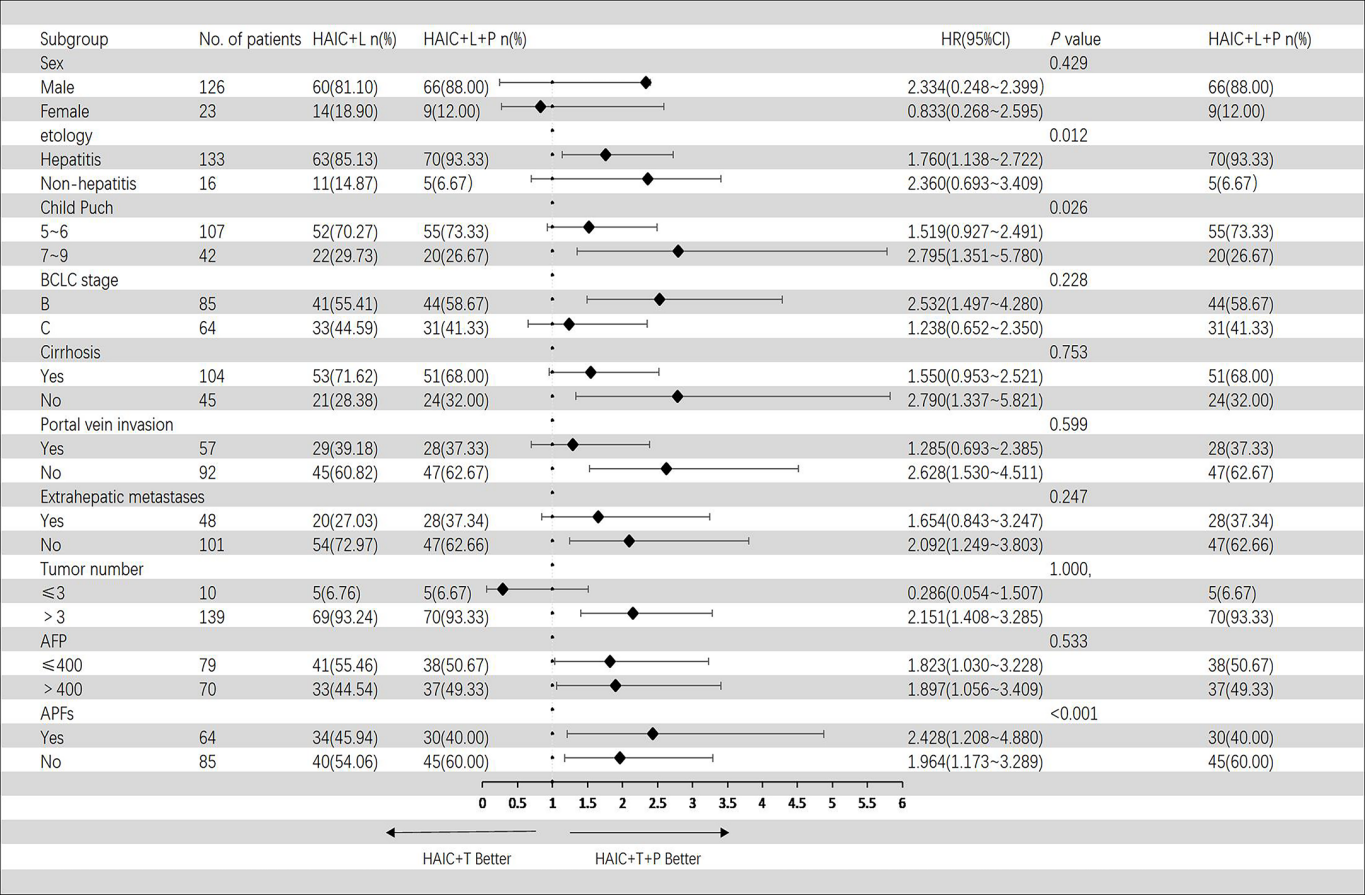
Forest plot of overall survival for subgroups in patients receiving the combination therapy of HAIC, lenvatinib plus PD-1 inhibitor, and HAIC plus lenvatinib therapy. HAIC, hepatic artery infusion chemotherapy; PD-1, programmed cell death-1.

In the Cox regression model of univariate analysis, therapy options (HAIC+T vs. HAIC+T+P), etiology of HCC, Child–Pugh score, and APFs were risk factors associated with overall survival mortality (*p* < 0.05) (**Table 3**). In multivariate analysis, therapy options (HAIC+T vs. HAIC+T+P) (hazard ratio (HR): 2.009; 95% CI: 1.332–3.029) (*p* = 0.001), Child–Pugh score (7–9 vs. 5–6) (HR: 1.612; 95% CI: 1.044–2.488) (*p* = 0.031), and APFs (no vs. yes) (HR: 0.468; 95% CI: 0.304–0.720) (*p* = 0.001) were significant predictors of OS (**Table 3**). **Figure 4** shows the subgroup analysis of OS. A significant benefit in OS was observed for HAIC+T+P in the following subgroups: hepatitis, Child–Pugh B, BCLC stage B, tumor number > 3, and AFP > 400 ng/ml.

**Table 3:** Univariable and multivariable Cox regression analyses for time to OS.

Characteristics	Univariable analysis			Multivariable analysis	
	HR (95% CI)	*p*-value		HR (95% CI)	*p*-value
Therapy options (HAIC+L vs. HAIC+L+P)	1.837 (1.226–2.753)	**0.003**		2.009 (1.332–3.029)	**0.001**
Age (years)	1.001 (0.982–1.019)	0.947			
Sex (male vs. female)	1.266 (0.705–2.274)	0.429			
Etiology of HCC and HBV (vs. others)	2.121 (1.178–3.817)	**0.012**		1.614 (0.888–3.032)	0.114
Child–Pugh score (7–9 vs. 5–6)	1.619 (1.058–2.477)	**0.026**		1.612 (1.044–2.488)	**0.031**
Cirrhosis (no vs. yes)	0.965 (0.772–1.206)	0.753			
BCLC stage (C vs. B)	1.285 (0.855–1.930)	0.228			
Portal vein invasion (yes vs. no)	1.115 (0.743–1.1.674)	0.599			
Tumor numbers (≤3 vs. >3)	1.000 (0.463–2.161)	1.000			
Largest tumor diameter (per cm)	1.050 (0.995–1.107)	0.077			
AFP (ng/ml; ≤400 vs. >400)	0.924 (0.722–1.184)	0.533			
Extrahepatic metastases (yes vs. no)	1.278 (0.844–1.936)	0.247			
APFs (no vs. yes)	0.466 (0.306–0.709)	**<0.001**		0.468 (0.304–0.720)	**0.001**

HR, hazard ratio; CI, confidence interval; HAIC, hepatic artery infusion chemotherapy; HBV, hepatitis B virus; BCLC, Barcelona Clinic Liver Cancer; AFP, alpha-fetoprotein; CA199, carbohydrate antigen 199; APFs, arterioportal fistulas.

The bold values denote statistical significance at P < 0.05 level.

**Figure 5:**
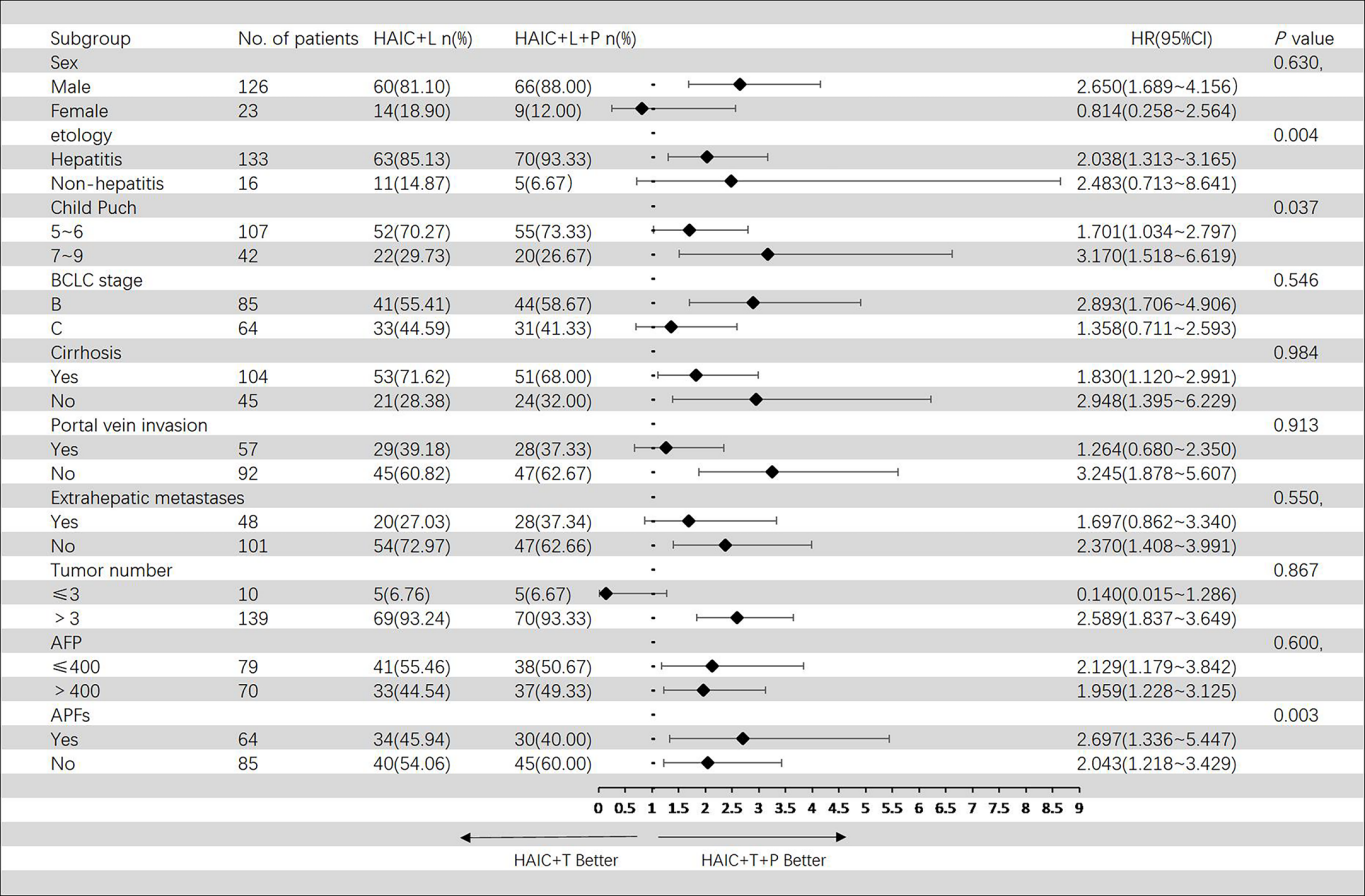
Forest plot of progression-free survival for subgroups in patients receiving the combination therapy of HAIC, lenvatinib plus PD-1 inhibitor, and HAIC plus lenvatinib therapy. HAIC, hepatic artery infusion chemotherapy; PD-1, programmed cell death-1.

In the Cox regression model of univariate analysis, therapy options (HAIC+T vs. HAIC+T+P), etiology of HCC, Child–Pugh score, and APFs were risk factors associated with progression-free survival mortality (*p* < 0.05) (**Table 4**). In multivariate analysis, therapy options (HAIC+T vs. HAIC+T+P) (HR: 2.175; 95% CI: 1.438–3.289) (*p* < 0.001), Child–Pugh score (7–9 vs. 5–6) (HR: 1.612; 95% CI: 1.044–2.492) (*p* = 0.031), and APFs (no vs. yes) (HR: 0.546; 95% CI: 0.354–0.841) (*p* = 0.006) were significant predictors of PFS (**Table 4**). **Figure 5** shows the subgroup analysis of PFS. A significant benefit in PFS was observed for HAIC+T+P in the following subgroups: male, hepatitis, Child–Pugh B, BCLC stage B, and tumor number > 3.

**Table 4:** Univariable and multivariable Cox regression analyses for time to PFS.

Characteristics	Univariable analysis		Multivariable analysis	
	HR (95% CI)	*p*-value	HR (95% CI)	*p*-value
Therapy options (HAIC+T vs. HAIC+T+P)	2.079 (1.382–3.126)	**<0.001**	2.175 (1.438–3.289)	**<0.001**
Age (years)	1.004 (0.985–1.022)	0.702		
Sex (male vs. female)	1.155 (0.643–2.073)	0.630		
Etiology of HCC and HBV (vs. others)	2.362 (1.310–4.259)	**0.004**	1.824 (0.981–3.395)	0.058
Child–Pugh score (7–9 vs.5–6)	1.573 (1.027–2.409)	**0.037**	1.612 (1.044–2.492)	**0.031**
Cirrhosis (no vs. yes)	0.998 (0.799–1.245)	0.984		
BCLC stage (C vs. B)	1.134 (0.753–1.708)	0.546		
Portal vein invasion (no vs. yes)	0.977 (0.649–1.473)	0.913		
Tumor numbers (≤3 vs. >3)	0.936 (0.433–2.025)	0.867		
Largest tumor diameter (per cm)	1.042 (0.988–1.099)	0.127		
AFP (ng/ml; ≤400 vs. >400)	0.908 (0.632–1.304)	0.600		
Extrahepatic metastases (yes vs. no)	1.136 (0.748–1.723)	0.550		
APFs (no vs. yes)	0.536 (0.352–0.814)	**0.003**	0.546 (0.354–0.841)	**0.006**

HR, hazards ratio; CI, confidence interval; HAIC, hepatic artery infusion chemotherapy; HBV, hepatitis B virus; BCLC, Barcelona Clinic Liver Cancer; AFP, alpha-fetoprotein; CA199, carbohydrate antigen 199; APFs, arterioportal fistulas.

The bold values denote statistical significance at P < 0.05 level.

Univariable and multivariable Cox regression analyses for OS and PFS between two groups before propensity matching are similar to those after propensity matching (**Supplementary Tables S2, 3**).

### Treatment safety

In the HAIC+T+P group, the most frequent TRAE was abdominal pain (53.33%); other TRAEs that occurred over 35% were creatinine increased (40.00%), hand-to-foot skin reaction (HFSR) (38.66%), and fatigue (36.00%). In the HAIC+T group, the most frequent TRAE was the same as that in HAIC+T+P group; other TRAEs that occurred over 35% were elevated serum AST or ALT (35.13%), creatinine increased (36.48%), and fatigue (37.83%).

In all grades of TRAEs, no significant between-group differences were revealed with regard to fever (*p* = 0.774), decreased appetite (*p* = 0.298), abdominal pain (*p* = 0.461), nausea/vomiting (*p* = 0.499), elevated serum AST or ALT (*p* = 0.817), thrombocytopenia (*p* = 0.209), gastrointestinal hemorrhage (*p* = 0.788), hyperbilirubinemia (*p* = 0.548), decreased albumin (*p* = 0.682), neutropenia (*p* = 0.442), increased creatinine (*p* = 0.659), liver abscess (*p* = 0.548), cholesteatoma (*p* = 0.638), cholecystitis (*p* = 0.548), HFSR (*p* = 0.250), skin rash (*p* =0.713), proteinuria (*p* = 0.250), fatigue (*p* = 0.816), bleeding (gingiva) (*p* = 0.745), and diarrhea (*p* = 0.137). However, the percentage of patients with hypertension in the HAIC+T+P group was significantly higher compared to the HAIC+T group (28.00% vs. 13.51%; *p* = 0.029).

In TRAEs with grade > 3 severity, no significant between-group differences were revealed with regard to hyperbilirubinemia (*p* = 0.689), hypertension (*p* = 0.622), skin rash (*p* =0.638), and fatigue (*p* = 0.548) (**Table 5**).

**Table 5:** TRAEs in the study population.

	All grades of TRAE			TRAE (more than grade 3)		
	HAIC+L+P (*n* = 75)	HAIC+L (*n* = 74)	*p*-value	HAIC+L+P (*n* = 75)	HAIC+L (*n* = 74)	*p*-value
Fever (*n* (%))	8 (10.66%)	9 (12.16%)	0.774			
Decreased appetite (*n* (%))	24 (32.00%)	18 (24.32%)	0.298			
Abdominal pain (%)	40 (53.33%)	35 (47.29%)	0.461			
Nausea/vomiting (no (%))	4 (5.33%)	6 (8.10%)	0.499			
Elevated serum AST or ALT (*n* (%))	25 (33.33%)	26 (35.13%)	0.817			
Thrombocytopenia (*n* (%))	16 (21.33%)	10 (13.51%)	0.209			
Gastrointestinal hemorrhage (*n* (%))	10 (13.33%)	11 (14.86%)	0.788			
Hyperbilirubinemia (*n* (%))	19 (25.33%)	22 (30.13%)	0.548	4 (5.33%)	2 (2.70%)	0.689
Albumin decreased (*n* (%))	13 (17.33%)	11 (14.86%)	0.682			
Neutropenia (*n* (%))	8 (10.66%)	11 (14.86%)	0.442			
Creatinine increased (*n* (%))	30 (40.00%)	27 (36.48%)	0.659			
Liver abscess (*n* (%))	1 (1.33%)	2 (2.70%)	0.548			
Cholesteatoma (*n* (%))	2 (2.66%)	3 (4.05%)	0.638			
Cholecystitis (*n* (%))	1 (1.33%)	2 (0.00%)	0.548			
Hypertension (*n* (%))	21 (28.00%)	10 (13.51%)	0.029	3 (4.00%)	1 (1.35%)	0.622
Hand–foot skin reaction (*n* (%))	29 (38.66%)	22 (29.72%)	0.250			
Skin rash (*n* (%))	16 (21.33%)	14 (18.91%)	0.713	2 (2.66%)	3 (4.05%)	0.638
Proteinuria (*n* (%))	21 (28.00%)	15 (20.27%)	0.250			
Fatigue (*n* (%))	27 (36.00%)	28 (37.83%)	0.816	1 (1.33%)	2 (4.00%)	0.548
Bleeding (gingiva; *n* (%))	4 (5.33%)	5 (6.75%)	0.745			
Diarrhea (*n* (%))	11 (14.66%)	18 (24.32%)	0.137			

HAIC, hepatic artery infusion chemotherapy; AST, aspartate aminotransferase; ALT, alanine aminotransferase.

No progressive deterioration of liver function indicators was associated with increasing treatment course, suggesting the safety of HAIC-based combination therapy treatment on liver function (**Supplementary Table S4**).

## Discussion

The efficacy and safety of the therapy for HCC refractory to TACE were investigated in our study. Our major findings were as follows: (1) compared to HAIC plus lenvatinib, combination therapy of HAIC, lenvatinib, and PD-1 inhibitors significantly improved OS, PFS, and DCR, demonstrating a better survival benefit; (2) therapy options (HAIC+T vs. HAIC+T+P) were significant predictors for OS and PFS; and (3) no significant between-group differences in TARE were revealed except for hypertension.

Providing appropriate subsequent therapy after TACE failure/refractoriness plays a key role in improving long-term outcomes in HCC patients. Following TACE failure or refractoriness, a variety of subsequent treatments have been investigated, including locoregional therapies, TKIs, PD-1 inhibitors, and combination therapies. For example, the study by Hsu et al. (9) reported the outcomes of HCC patients with TACE failure/refractoriness receiving HAIC with a modified FOLFOX regimen. The median OS and PFS were up to 9 and 3.7 months, respectively. Notably, Klompenhouwer et al. (26) administered the effectiveness of transarterial radioembolization for HCC with drug-eluting beads after TACE failure/refractoriness. The median OS was up to 14.8 months. Xu et al. (27) performed a retrospective study that reported the clinical outcomes of iodine-125 seed implantation for therapy of HCC with TACE failure/refractoriness. The median TTP was 8.8 months, and the ORR was 90.5%. Arizumi et al. (28) and Ogasawara et al. (13) compared the therapy efficacy of continued TACE and sorafenib monotherapy for HCC patients with TACE failure/refractoriness, notably. Both studies demonstrated that, compared with continued TACE, sorafenib monotherapy demonstrated a superior outcome. Recently, Zheng et al. (19) explored the potential of TACE combined with sorafenib and immune checkpoint inhibitor therapy for patients after TACE failure/refractoriness. A triple-combination treatment group showed significantly greater treatment efficacy than the TACE plus sorafenib group, with better DCR (81.82% vs. 55.17%; *p* = 0.046), longer median PFS (16.26 vs. 7.30 months) (*p* < 0.001), and longer median OS (23.3 vs. 13.8 months) (*p* = 0.012). Additionally, these HCC patients may be cured only by conversion resection. TACE-based combination therapy is playing an increasingly important role in tumor downstaging and translational surgical resection of unresectable hepatocellular carcinoma because of its better tumor response rate and better survival benefit (29). Romic et al. reported that this translational surgical resection resulted in better clinical outcomes and a better survival prognosis (30).

HAIC is a local treatment with fewer systemic adverse effects and high drug concentrations in the liver (31). Although there is emerging evidence that HAIC is a safe and effective therapeutic approach for TACE-refractory HCC (32, 33), treatment response remains limited, and survival benefits are still unsatisfactory (34). Combination therapy for TACE-refractory HCC has been explored (18). For example, several studies have shown that the combination of HAIC plus TKI resulted in better survival outcomes compared to TKI or HAIC monotherapy (32, 35, 36). The OS reported in our study was 9.0 months, which was lower compared to previous clinical trials (37, 38). This difference may be explained by the poor baseline characteristics of the patients. As a result of the epidemiological differences, TACE is applied from BCLC stages A to C based on the Chinese guidelines (2019 edition) (39), with a more severe tumor burden (93.29% of patients had more than three tumors) and the fact that they were in the late stages of BCLC (32.22% of patients had extrahepatic metastases, while 38.25% had vascular invasion), reflecting a poor prognosis. In addition, prior studies have predominantly included newly diagnosed HCC, while this study involved nearly all patients who had already received multiple TACE treatments, which may compromise the arteries supplying tumors (40). Therefore, it affects the effectiveness of HAIC treatment, resulting in a poor survival prognosis (33).

In this work, patients who received HAIC in combination with lenvatinib and PD-1 inhibitors had a median OS of 16.0 months, consistent with a previous work that showed an OS of 15.8 months (41). In the present study, the median PFS for these patients was 11.0 months, higher than the 6.5-month PFS observed in the previous study (41). This inconsistency in PFS may be due to poor clinical baseline characteristics. This previous trial included a larger proportion (20.00%) of patients with inferior vena cava tumor thrombosis, whereas our study did not include such patients. Inferior vena cava tumor thrombosis is known to increase the risk of death and has a devastating effect on long-term survival (42). In this study, our results showed prolonged OS from 9 to 16 months in patients with TACE refractory receiving HAIC in combination with lenvatinib plus a PD-1 inhibitor compared to those receiving only HAIC in combination with lenvatinib. This result may be due to a trend toward better DCR and a relatively longer PFS in patients treated with HAIC+T+P rather than HAIC+T. The efficacy and safety of the triple therapy suggested in our study may be explained by these findings as follows: In addition to releasing proangiogenic cytokines, HAIC-induced hypoxia promotes immune cell death as well. Furthermore, these factors stimulated tumor angiogenesis by modulating immune function within the tumor microenvironment (21). In addition, first-line TKIs such as lenvatinib can increase PD-L1 expression in tumors and promote immune cell infiltration into tumors (43). Combining TKIs with PD-1 inhibitors can produce unique immunomodulatory effects that can overcome the challenges of low response rates and TACE resistance (44–46). Therefore, it is expected that combining HAIC with first-line TKIs plus PD-1 inhibitors could improve tumor response rates and be effective in improving the prognosis of our study patients.

Based on subgroup analyses of hepatitis, Child–Pugh B, BCLC stage B, tumor number > 3, and AFP > 400 ng/ml, HAIC+T+P contributed to a better OS, whereas the other subgroups did not. Similar results have been reported in advanced HCC (47–49). The application of PD-1 inhibitors or insufficient sample size may be responsible for this. Using Cox multivariate regression analysis, we found that treatment options were an independent risk factor for OS and PFS. Consequently, we concluded that PD-1 inhibitors might improve these patients’ clinical outcomes.

This study had acceptable adverse events. The TRAEs were more prevalent in the HAIC+T+P group than in the HAIC+T group. These TRAEs, including increased creatinine, hand-to-foot skin reactions, fatigue, etc., likely contributed to the side effects of PD-1 inhibitors (50–52). However, these adverse events were primarily grade 1 or 2 TAREs that could be relieved or eliminated by treating the symptoms or adjusting the dose. Therefore, we believe that HAIC combined with lenvatinib plus PD-1 inhibitor therapy for these patients was acceptable and feasible.

The present study has several limitations. First, the treatment options in this study were based on physician and patient preferences, which created a selection bias in the study population. Second, this is a single-center retrospective study with inherent drawbacks, which limit the ability to draw general conclusions. Third, this is a small sample study with a heterogeneous etiology of cirrhosis. Previous studies have shown that the etiology of cirrhosis is related to the efficacy of TKIs (53, 54). Further studies are necessary to perform a subgroup analysis of the etiology of cirrhosis.

In conclusion, in HCC patients with refractory to TACE, combination therapy consisting of HAIC, lenvatinib, and PD-1 inhibitors may be associated with improved OS and PFS, and this regimen deserves to be considered an optimization approach. Our findings should be validated by large samples and randomized controlled trials.

## Data availability statement

The original contributions presented in the study are included in the article/**Supplementary Material**. Further inquiries can be directed to the corresponding author.

## Ethics statement

This retrospective study was endorsed by the ethics committee of Fujian Medical University Union Hospital, Fuzhou, China.

## Author contributions

Concept and design of the study: J-YH. Acquisition of the data: L-YY and L-WL. Analysis and interpretation of the data: L-WL and KK. Drafting of the manuscript: L-WL and RC. Critical revision of the manuscript for important intellectual content: J-YH, RC, and L-WL. Administrative, technical, or material support and study supervision: L-YY and L-WL. All authors contributed to the article and approved the submitted version.
