# Supplementary material for: Efficacy and safety of hepatic artery infusion chemotherapy combined with tyrosine kinase inhibitors plus programmed death-1 inhibitors for hepatocellular carcinoma refractory to transarterial chemoembolization
Source: Front Oncol. 2023 May 3;13:1178428. doi: 10.3389/fonc.2023.1178428 (PMC10189040; doi:10.3389/fonc.2023.1178428)
Supplement: Supplementary file 1 [file DataSheet_1.pdf]

Supplementary material Table 1. Treatment response as assessed by imaging features according to the mRECIST criteria in two groups before and after PSM analysis

Curative effect	Before PSM HAIC+L+P (n=75)	HAIC+L (n=74)	*P value*	After PSM HAIC+L+P (n=48)	HAIC+L (n=48)	*P value*
Complete response (CR)	1(1.33%)	1(1.35%)		1(2.08%)	1(2.08%)	
Partial response (PR)	33(44.00%)	24(32.43%)		21(43.75%)	15(31.25%)	
Stable disease (SD)	25(33.33%)	21(28.37%)		16(33.33%)	13(25.00%)	
Progressive disease (PD)	16(21.33%)	28(37.83%)		10(20.83%)	19(39.58%)	
Overall response rate (ORR)	34(45.33%)	25(33.78%)	0.146	22(45.83%)	16(33.33%)	0.210
Disease control rate (DCR)	59(78.66%)	46(62.16%)	0.027	38(79.16%)	29(60.41%)	0.045

Abbreviations: mRECIST, Modified Response Evaluation Criteria in Solid Tumours; HAIC, hepatic artery infusion chemotherapy. PSM, propensity score matching.

Supplementary material Table 2. Univariable and multivariable Cox regression analyses for time to OS before and after PSM analysis

Characteristics	Before PSM Univariable analysis		Before PSM Multivariable analysis		After PSM Univariable analysis		After PSM Multivariable analysis	
	HR (95% CI)	*P value*	HR (95% CI)	*P value*	HR (95% CI)	*P value*	HR (95% CI)	*P value*
therapy options		**0.003**		**0.001**		**0.030**		**0.011**
HAIC+L+P	reference		reference		reference		reference	
HAIC+L	1.837(1.226-2.753)		2.009(1.332-3.029)		1.740(1.056-2.867)		1.931(1.166-3.198)	
Age (years)	1.001(0.982-1.019)	0.947			0.990(0.967-1.014)	0.413		
Sex		0.429				0.550		
Male	1.266(0.705-2.274)				1.254(0.596-2.639)			
Female	reference				reference			
etiology of HCC		**0.012**		0.114		0.376		
HBV	2.121(1.178-3.817)		1.614(0.888-3.032)		1.516(0.604-3.803)			
Others	reference		reference		reference			
Child Pugh score		**0.026**		**0.031**		0.785		
5-6	reference		reference		reference			
7-9	1.619(1.058-2.477)		1.612(1.044-2.488)		1.082(0.613-1.912)			
Cirrhosis		0.753				0.958		
Yes	reference				reference			
No	0.965(0.772-1.206)				0.985(0.551-1.760)			
BCLC stage		0.228				0.211		
B	reference				reference			
C	1.285(0.855-1.930)				1.376(0.835-2.267)			
Portal vein invasion		0.599				0.135		
Yes	1.115(0.743-1.1.674)				1.470(0.887-2.436)			
No	reference				reference			
Tumor numbers		1.000				0.238		
≤3	reference				reference			
＞3	1.000(0.463-2.161)				1.671(0.712-3.922)			
AFP (ng/ml)		0.533						
>400	reference				reference			
<400	0.924(0.722-1.184)				0.828(0.503-1.364)	0.459		
Extrahepatic metastases		0.247				0.390		
Yes	1.278(0.844-1.936)				1.253(0.750-2.094)			
No	reference				reference			
APFs		**<0.001**		**0.001**		**0.016**		**0.006**
Yes	reference		reference		reference		reference	
No	0.466(0.306-0.709)		0.468(0.304-0.720)		0.534(0.320-0.891)		0.486(0.290-0.815)	
Largest tumor size (cm)	1.050(0.995-1.107)	0.077			1.074(1.002-1.153)	0.065		

Abbreviations: HR, hazard ratio; CI, confidence interval; HAIC, hepatic artery infusion chemotherapy; HBV, hepatitis B virus; BCLC, Barcelona Clinic Liver Cancer; PVTT, portal vein tumor thrombosis; AFP, alpha-fetoprotein; CA199, carbohydrate antigen 199; APFs, arterioportal fistulas. PSM, propensity score matching.

Supplementary material Table 3. Univariable and multivariable cox regression analyses for time to PFS before and after PSM analysis

Characteristics	Before PSM				After PSM			
	Univariable analysis		Multivariable analysis		Univariable analysis		Multivariable analysis	
	HR (95% CI)	*P value*	HR (95% CI)	*P value*	HR (95% CI)	*P value*	HR (95% CI)	*P value*
therapy options		**<0.001**		**<0.001**		**0.037**		**0.037**
HAIC+L+P	2.079(1.382-3.126)		2.175(1.438-3.289)		1.704(1.034-2.807)		1.704(1.034-2.807)	
HAIC+L)	reference				reference			
Age (years)	1.004(0.985-1.022)	0.702			0.992(0.969-1.016)	0.506		
Sex		0.630				0.836		
Male	1.155(0.643-2.073)				1.082(0.514-2.277)			
Female	reference				reference			
etiology of HCC		**0.004**		0.058		0.237		
HBV	2.362(1.310-4.259)		1.824(0.981-3.395)		1.743(0.693-4.384)			
Others	reference				reference			
Child Pugh score		**0.037**		**0.031**		0.983		
5-6	reference		reference		reference			
7-9	1.573(1.027-2.409)		1.612(1.044-2.492)		1.006(0.569-1.779)			
Cirrhosis		0.984				0.722		
Yes	reference				reference			
No	0.998(0.799-1.245)				0.986(0.622-1.287)			
BCLC stage		0.546				0.457		
B	reference				reference			
C	1.134(0.753-1.708)				1.209(0.734-1.991)			
Portal vein invasion		0.913				0.231		
Yes	reference				reference			
No	0.977(0.649-1.473)				0.964(0.521-1.265)			
Tumor numbers		0.867				0.367		
≤3	0.936(0.433-2.025)				0.977(0.633-2.445)			
＞3	reference				reference			
AFP (ng/ml)		0.600				0.354		
>400	reference				reference			
<400	0.908(0.632-1.304))				0.791(0.0.482-1.298)			
Extrahepatic metastases		0.550				0.758		
Yes	1.136(0.748-1.723)				1.084(0.648-1.814)			
No	reference				reference			
APFs		**0.003**		**0.006**		0.055		
Yes	reference		reference		reference			
No	0.536(0.352-0.814)		0.546(0.354-0.841)		0.607(0.365-1.011)			
Largest tumor size (cm)	1.042(0.988-1.099)	0.127			1.062(0.992-1.138)	0.085		

Abbreviations: HR, hazards ratio; CI, confidence interval; HAIC, hepatic artery infusion chemotherapy; HBV, hepatitis B virus; BCLC, Barcelona Clinic Liver Cancer; PVTT, portal vein tumor thrombus; AFP, alpha-fetoprotein; CA199, carbohydrate antigen 199; APFs, arterioportal fistulas. PSM, propensity score matching.

Supplementary material Table 4. Comparison of Child Pugh classification before treatment and after different numbers HAIC courses treatment in the HAIC+L+P group and HAIC+L group.

treatment course	Variable	HAIC+L+P Before therapy	After therapy	*P value*	HAIC+L+ Before therapy	After therapy	*P value*
2 HAIC course	Child Pugh classification A	55(73.33%)	53(70.66%)	0.716	52(70.27%)	51(68.91%)	0.858
	B	20(26.67%)	22(29.34%)		22(29.73%)	23(31.09%)	
3 HAIC course	Child Pugh classification A	55(73.33%)	48(64.00%)	0.218	52(70.27%)	54(72.97%)	0.715
	B	20(26.67%)	27(36.00%)		22(29.73%)	20(27.03%)	
3 HAIC course	Child Pugh classification A	55(73.33%)	50(66.66%)	0.373	52(70.27%)	55(74.32%)	0.582
	B	20(26.67%)	25(33.37%)		22(29.73%)	19(25.68%)	
4 HAIC course	Child Pugh classification A	55(73.33%)	57(76.00%)	0.707	52(70.27%)	50(67.56%)	0.722
	B	20(26.67%)	18(24.00%)		22(29.73%)	24(32.44%)	
5 HAIC course	Child Pugh classification A	55(73.33%)	52(69.33%)	0.588	52(70.27%)	48(64.86%)	0.535
	B	20(26.67%)	23(30.67%)		22(29.73%)	16(35.14%)	
6 HAIC course	Child Pugh classification A	55(73.33%)	50(66.66%)	0.373	52(70.27%)	56(75.67%)	0.459
	B	20(26.67%)	25(33.37%)		22(29.73%)	18(24.33%)	
7 HAIC course	Child Pugh classification A	55(73.33%)	51(68.00%)	0.473	52(70.27%)	50(67.56%)	0.722
	B	20(26.67%)	24(32.00%)		22(29.73%)	24(32.44%)	

Abbreviations: mRECIST, Modified Response Evaluation Criteria in Solid Tumours; HAIC, hepatic artery infusion chemotherapy; PD-1, programmed death-1.

Supplementary material Figure legends

Supplementary material Figure 1. Kaplan–Meier analysis of overall survival in patients receiving the combination therapy of HAIC, lenvatinib plus PD-1 inhibitor, and HAIC plus lenvatinib therapy after PSM analysis. HAIC, hepatic artery infusion chemotherapy; PD-1, programmed cell death-1; PSM, propensity score matching.

Supplementary material Figure 2. Kaplan–Meier analysis of progression-free survival in patients receiving the combination therapy of HAIC, lenvatinib plus PD-1 inhibitor, and HAIC plus lenvatinib therapy after PSM analysis. HAIC, hepatic artery infusion chemotherapy; PD-1, programmed cell death-1; PSM, propensity score matching.

Supplementary material Figure 3. Kaplan–Meier analysis of overall survival in patients receiving the numbers of HAIC courses. HAIC, hepatic artery infusion chemotherapy; PD-1, programmed cell death-1.

Supplementary material Figure 4. Kaplan–Meier analysis of progression-free survival in patients receiving the numbers of HAIC courses. HAIC, hepatic artery infusion chemotherapy; PD-1, programmed cell death-1.

Supplementary material Figure 5 This magnetic resonance (MR) images showed the imaging feature of 58-year-old men with HCC accompanied by portal vein tumor thrombosis (PVTT), which classified as the type 3 of portal vein tumor thrombosis using Cheng’s PVTT classification. T2 weighted imaging (A), contrast-enhanced MR scan (B) showed a giant tumor had a tumour thrombus in the left branch of the portal vein before HAIC combined with lenvatinib therapy; T2 weighted imaging (C), contrast-enhanced scan MR scan (D) showed a giant tumor with PVTT was completely inactive and achieved CR according to the mRECIST criteria after 5 courses of HAIC combined with lenvatinib therapy. HAIC, hepatic artery infusion chemotherapy; CR, complete response; mRECIST, the Modified Response Evaluation Criteria in Solid Tumors.

Supplementary material Figure 6 This magnetic resonance (MR) images showed the imaging feature of 56-year-old woman with HCC accompanied by portal vein tumor

thrombosis (PVTT) and superior mesenteric vein tumor thrombosis (SMVTT), which classified as the type 4 of portal vein tumour thrombosis using Cheng's PVTT classification. T2 weighted imaging (A) (B), contrast-enhanced MR scan (C) showed multiple liver tumors had a tumor thrombus in portal vein and superior mesenteric vein before HAIC combined with lenvatinib plus PD-1 inhibitors therapy; T2 weighted imaging (D) (E), contrast-enhanced MR scan (F) showed multiple liver tumors with PVTT and SMVCT was partially active and achieved PR according to the mRECIST criteria after 3 courses of HAIC combined with lenvatinib plus PD-1 inhibitors therapy; T2 weighted imaging (G) (H), contrast-enhanced MR scan (I) showed multiple liver tumors with PVTT and SMVTT was completely inactive and achieved CR according to the mRECIST criteria after 6 courses of HAIC combined with lenvatinib plus PD-1 inhibitors therapy. The previously blocked portal vein and superior mesenteric vein due to PVTT and SMVTT also restores blood flow. HAIC, hepatic artery infusion chemotherapy; CR, complete response; mRECIST, the Modified Response Evaluation Criteria in Solid Tumors.
