# Supplementary figures and images for: Efficacy and safety of hepatic artery infusion chemotherapy combined with tyrosine kinase inhibitors plus programmed death-1 inhibitors for hepatocellular carcinoma refractory to transarterial chemoembolization
Source: Front Oncol. 2023 May 3;13:1178428. doi: 10.3389/fonc.2023.1178428 (PMC10189040; doi:10.3389/fonc.2023.1178428)

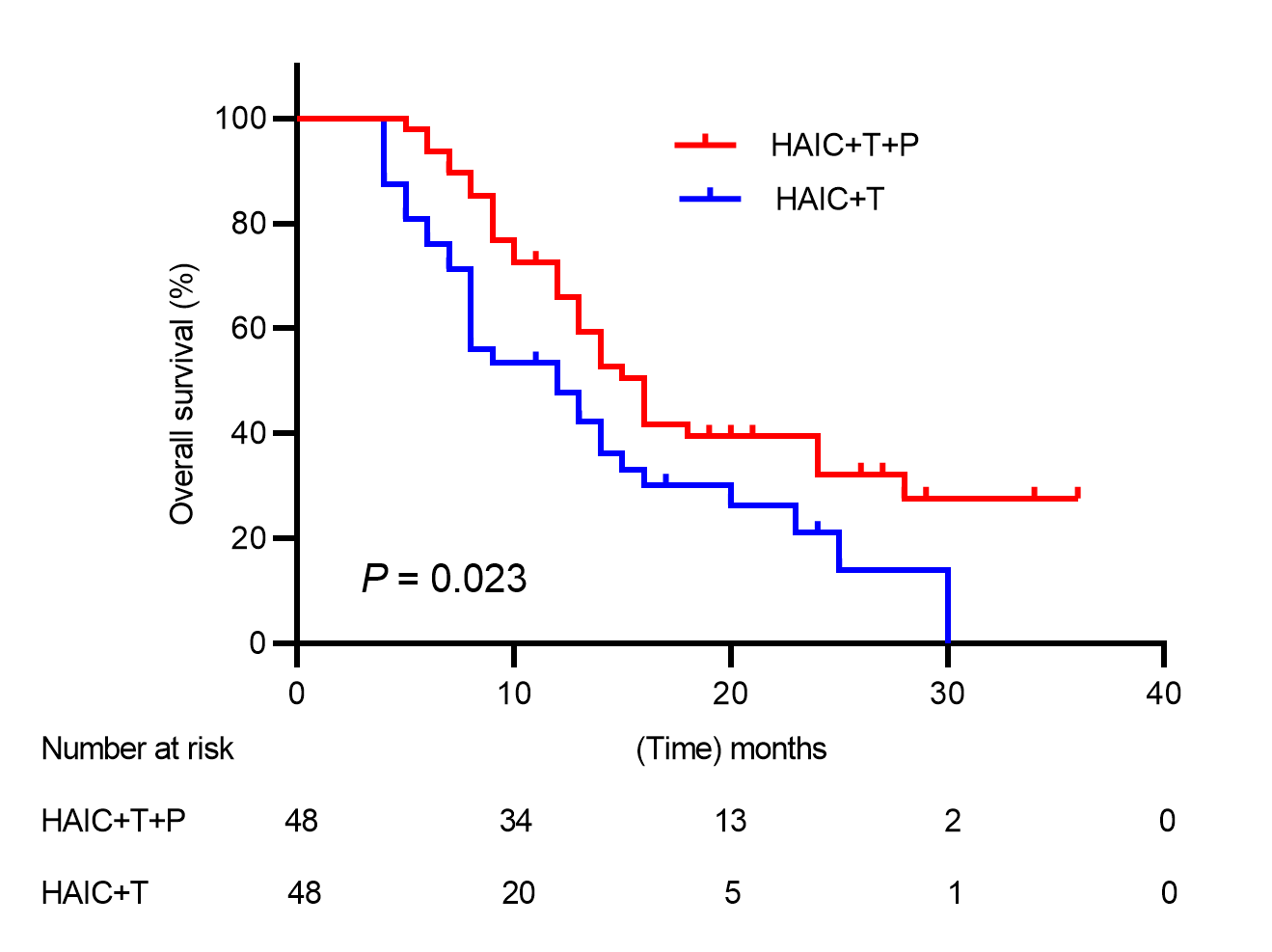

Supplement: Supplementary file 2 [file Image_1.tif]

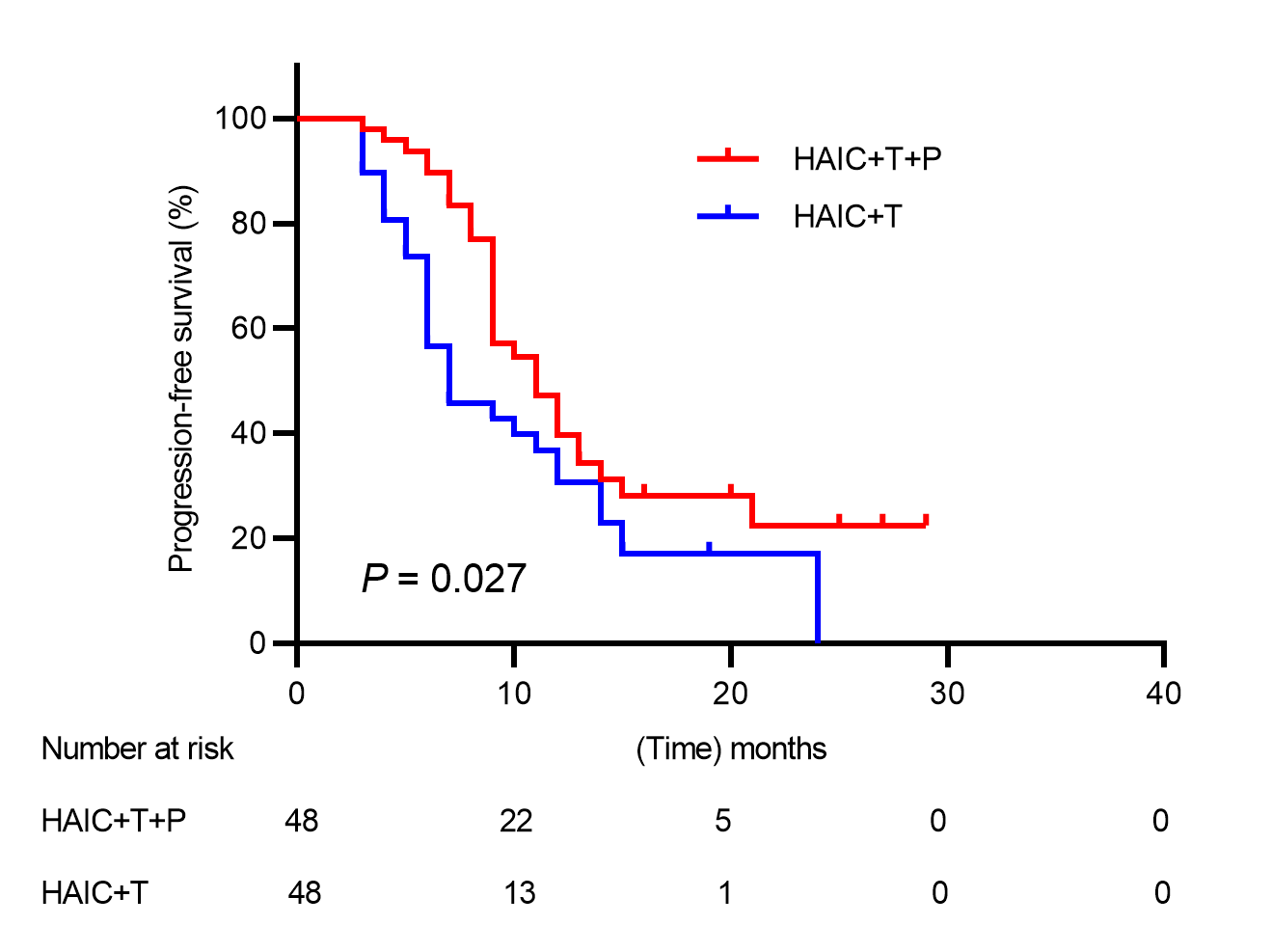

Supplement: Supplementary file 3 [file Image_2.tif]

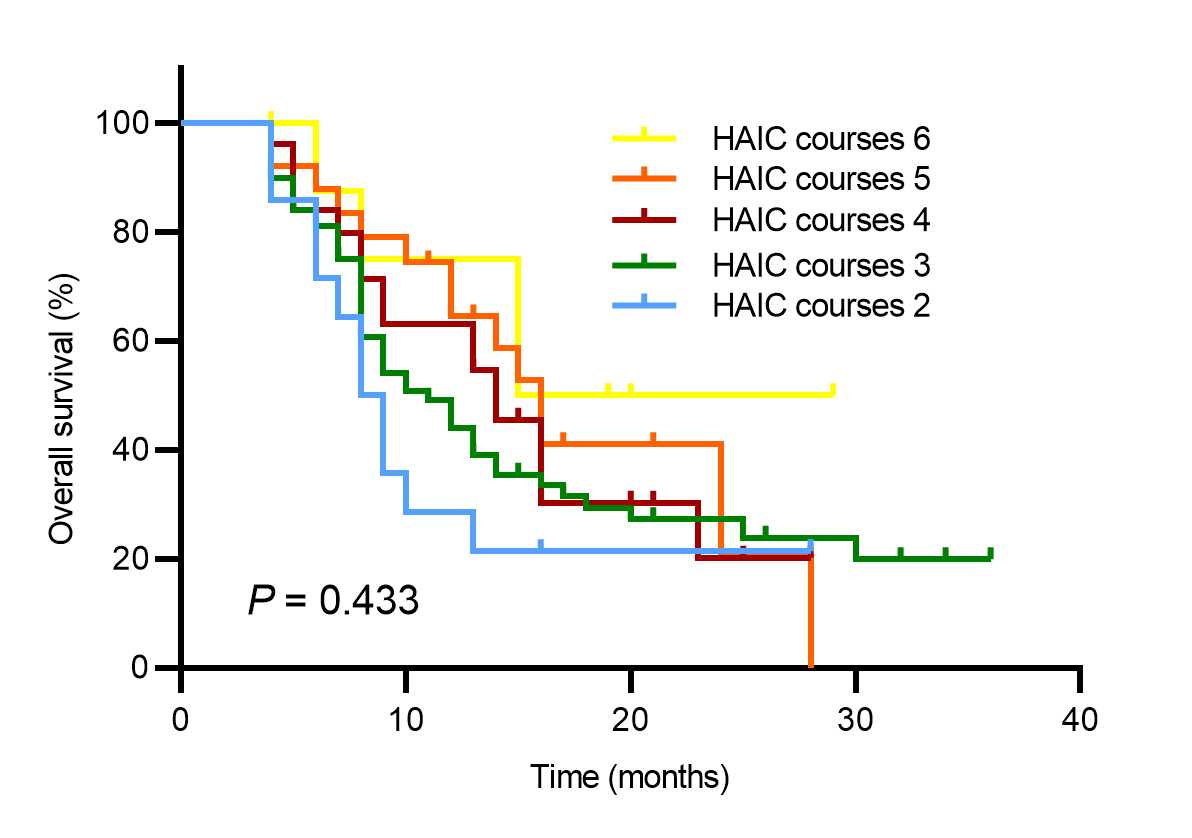

Supplement: Supplementary file 4 [file Image_3.tif]

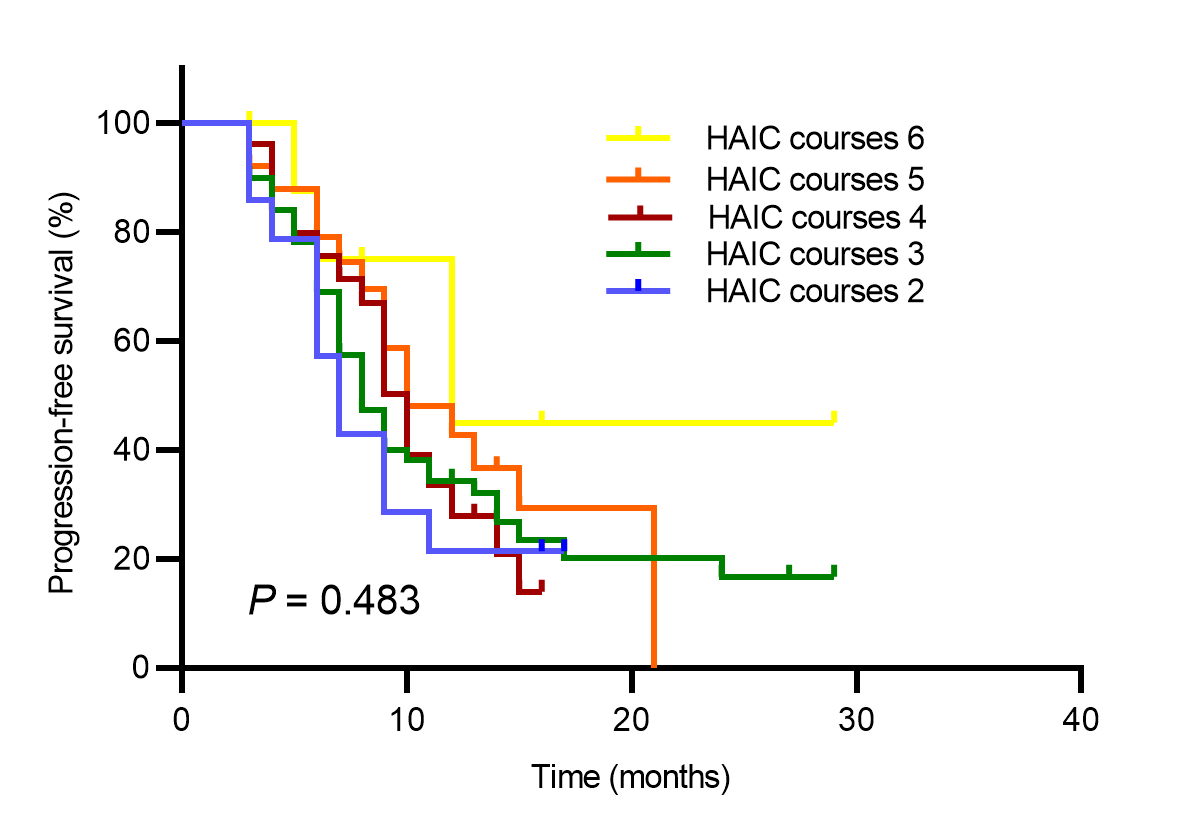

Supplement: Supplementary file 5 [file Image_4.tif]

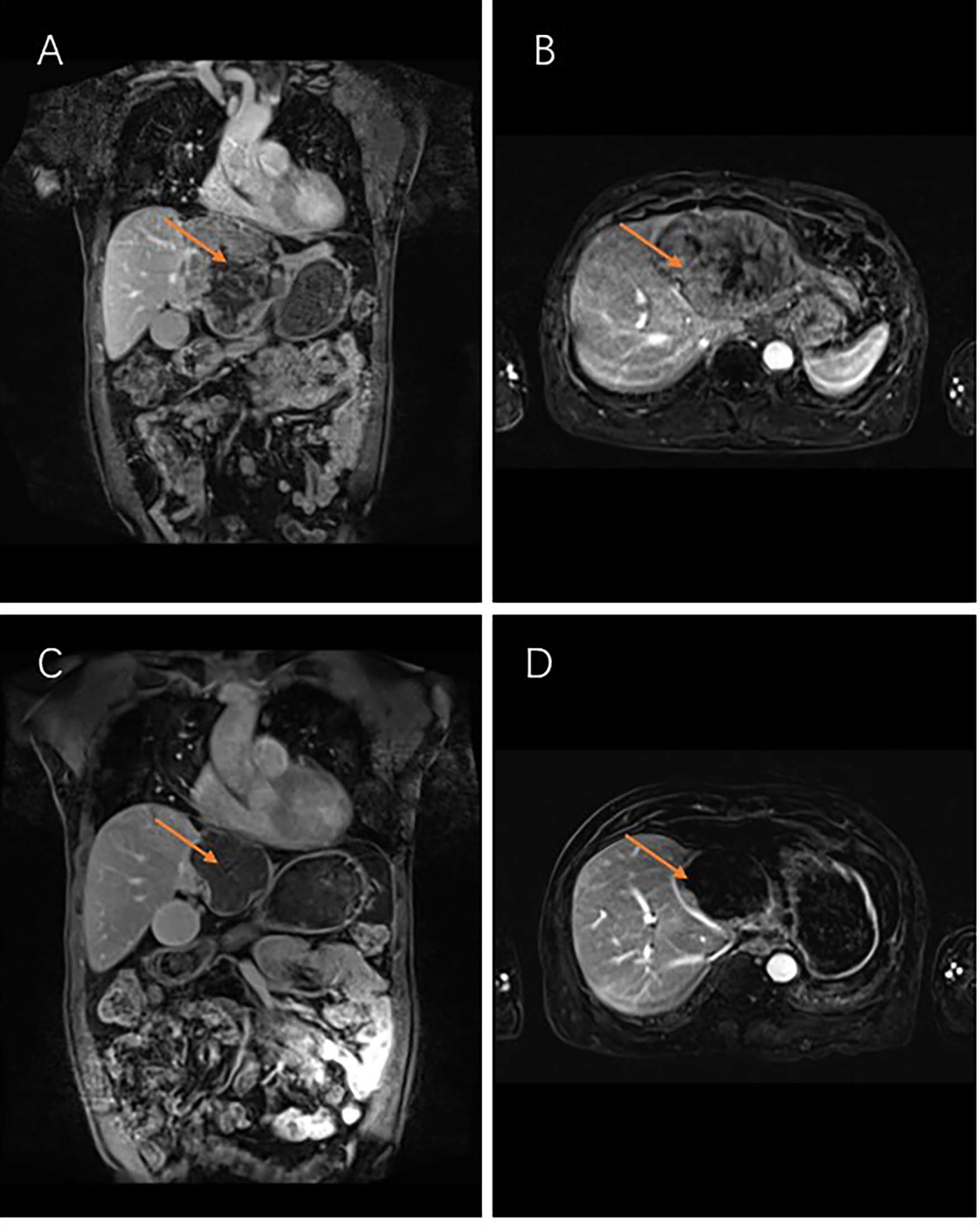

Supplement: Supplementary file 6 [file Image_5.tif]

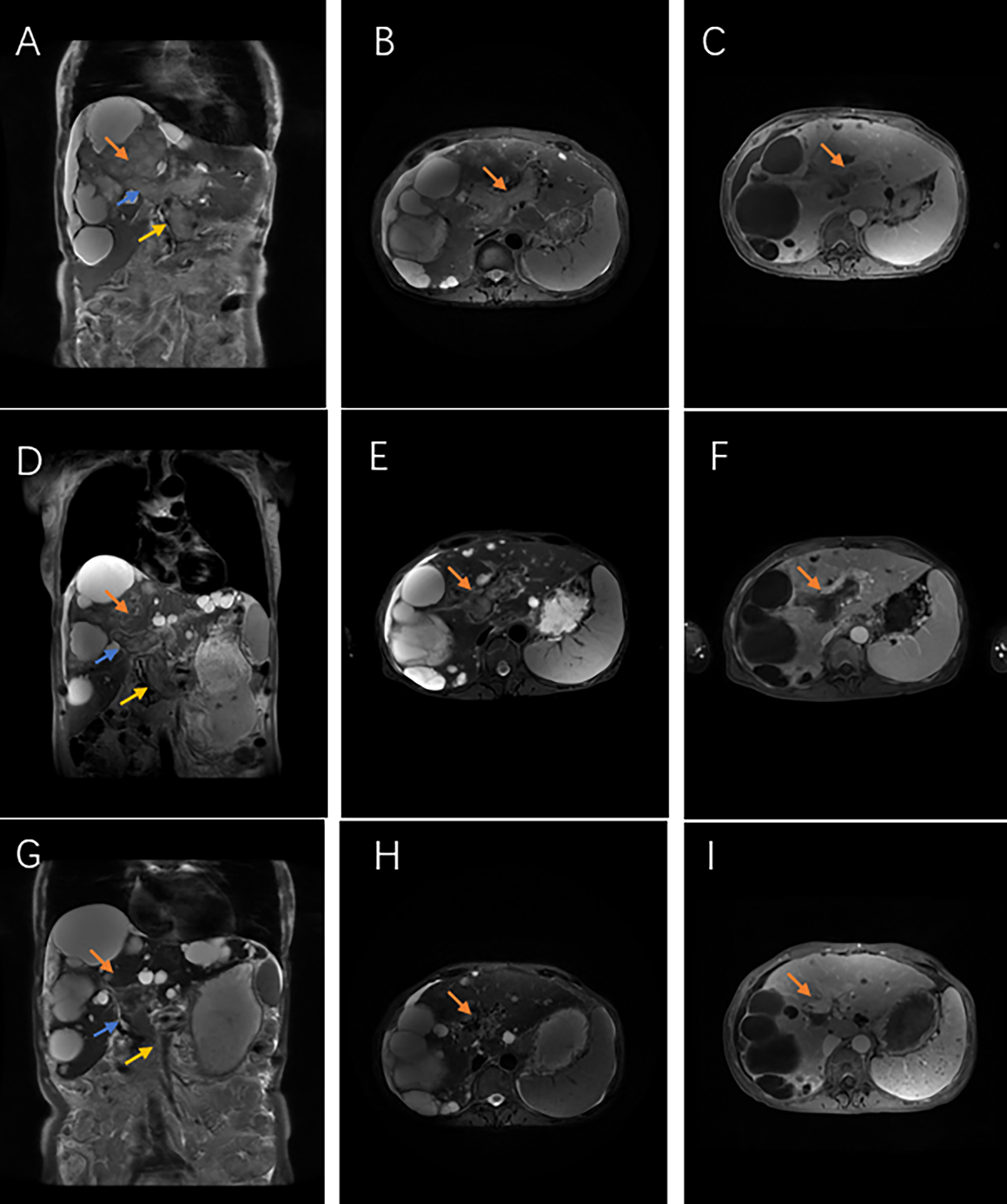

Supplement: Supplementary file 7 [file Image_6.tif]
